# Disease–disease interactions: molecular links of neurodegenerative diseases with cancer, viral infections, and type 2 diabetes

**DOI:** 10.1186/s40035-025-00507-3

**Published:** 2025-10-17

**Authors:** Yuxi Lin, Je Min Yoo, Yan Li, Yunseok Heo, Masaki Okumura, Hyung-Sik Won, Michele Vendruscolo, Mi Hee Lim, Young-Ho Lee

**Affiliations:** 1 Biopharmaceutical Research Center, Korea Basic Science Institute (KBSI), Ochang, Chungbuk 28119 Republic of Korea; 2 Chaperone Ventures LLC., Los Angeles, CA 90010 USA; 3 Research Institute of Biomedical and Health Science, Konkuk University, Chungju, Chungbuk 27478 Republic of Korea; 4 Frontier Research Institute for Interdisciplinary Sciences, Tohoku University, Sendai, Miyagi 980-8578 Japan; 5 BK21 Project Team, Department of Applied Life Science, Graduate School, Konkuk University, Chungju, Chungbuk 27478 Republic of Korea; 6 Centre for Misfolding Diseases, Yusuf Hamied Department of Chemistry, University of Cambridge, Cambridge, CB2 1EW UK; 7 Department of Chemistry, Korea Advanced Institute of Science and Technology (KAIST), Daejeon, 34141 Republic of Korea; 8 Bio-Analytical Science, University of Science and Technology (UST), Daejeon, 34113 Republic of Korea; 9 Graduate School of Analytical Science and Technology, Chungnam National University, Daejeon, 34134 Republic of Korea; 10 Department of Systems Biotechnology, Chung-Ang University (CAU), Gyeonggi, 17546 Republic of Korea; 11 Graduate School of Life Sciences, Tohoku University, Sendai, Miyagi 980-8577 Japan

**Keywords:** Disease–disease interactions, Neurodegenerative diseases, Cancer, Infectious diseases, Diabetes, Underlying mechanisms

## Abstract

Neurodegenerative disorders, notably Alzheimer’s and Parkinson’s diseases, are unified by progressive neuronal loss and aberrant protein aggregation. Growing evidence indicates that these conditions are linked to cancer, infectious diseases, and type 2 diabetes through convergent molecular processes. In this review, we examine the mechanistic foundations of these links, focusing on shared features such as protein misfolding and aggregation, chronic inflammation, and dysregulated signalling pathways. We integrate cellular, animal, and human data to illustrate how pathogenic proteins may influence one another through cross-seeding and co-aggregation, and assess the implications of such interactions for disease susceptibility, progression, and treatment response. Understanding these underlying mechanisms may provide a conceptual framework for developing therapeutic approaches that target the molecular basis of multiple complex disorders.

## Background

Many chronic disorders share common molecular patterns that transcend clinical boundaries [1–7]. Disease processes initiated by distinct triggers in different tissues often converge on a limited set of biochemical responses that determine cell fate. Such convergence suggests that disparate diseases may not only share molecular hallmarks, but also influence one another through the systemic circulation of pathogenic factors, including cytokines, hormones, extracellular vesicles, and misfolded-protein seeds, and the modulation of overlapping signalling networks. Understanding the extent to which these interactions are governed by biophysical principles, such as the competition for limited stress-response capacity or the templated aggregation of misfolded proteins, provides powerful opportunities to uncover unifying mechanisms underlying disease progression and comorbidity [8–11].

This framework yields testable mechanisms linking diseases via shared molecules, overlapping signalling networks, and common dysregulation. For example, activation of the nucleotide-binding domain leucine-rich repeat and pyrin domain containing receptor protein 3 (NLRP3) inflammasomes has been implicated in both COVID-19 and Parkinson’s disease (PD), indicating a shared inflammatory process [12, 13]. Similarly, the phosphoinositide 3-kinases/Akt/mammalian target of rapamycin (PI3K/Akt/mTOR) signalling pathway is involved in both cancer and Alzheimer’s disease (AD), although it is differentially regulated under these conditions [14]. Similarly, protein misfolding events occur for amylin in type 2 diabetes (T2D) and amyloid-β (Aβ) in AD, reflecting a convergence of pathogenic mechanisms [15].

Investigating these connections at the molecular and cellular levels reveals common biochemical pathways that may influence the onset, progression, and therapeutic response of different diseases. For example, protein cross-seeding, where the misfolding and aggregation of proteins associated with one disease catalyses similar pathological processes in another, illustrates the crosstalk between apparently unrelated conditions. This mechanism has been observed not just with neurodegenerative diseases (NDDs) among themselves [11, 16], but also with cancer [9, 17, 18], infectious diseases [8, 19, 20], and diabetes [11, 21].

Insights from network-based approaches in human disease may have far-reaching implications for public health and therapeutic innovation. By exploring the mechanisms underpinning disease-disease interactions, opportunities may arise for novel interventions, including the repurposing of drugs originally designed for unrelated conditions [22–27]. For instance, anticancer agents have shown promise in targeting pathways implicated in neurodegeneration [28–30], while antidiabetic drugs are being investigated for their neuroprotective properties [31, 32]. By leveraging such cross-disease insights, integrated strategies can be developed that not only improve individual patient outcomes but also address the growing burden of comorbid diseases.

**Fig. 1:**
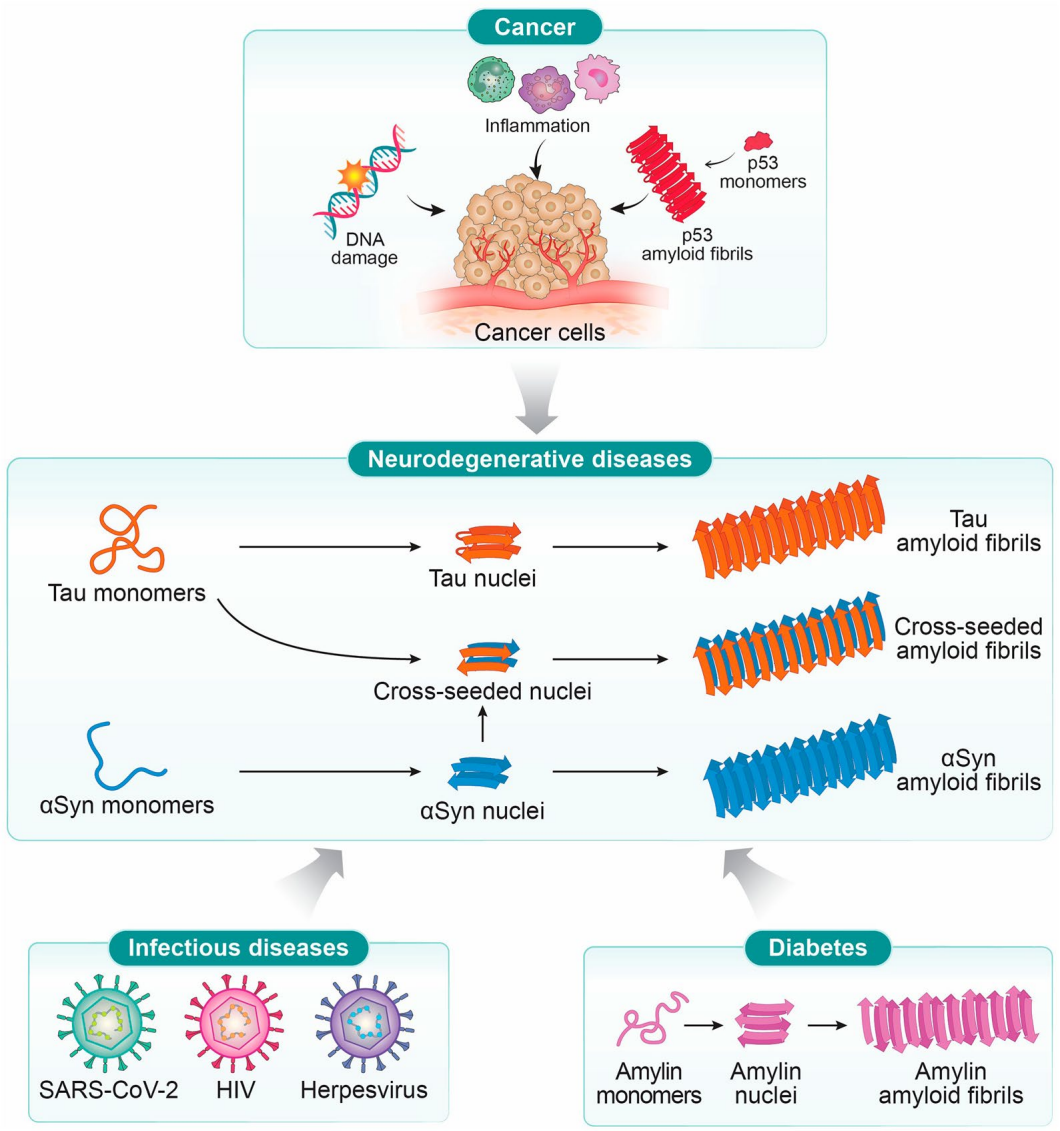
Neurodegenerative diseases that may be affected by infectious diseases, cancer, and diabetes. Some examples of key molecular species associated with this phenomenon are illustrated and described in the main text

In this review, we discuss the complex connections between NDDs and a range of other conditions, including infectious diseases, cancer, and diabetes, drawing from recent studies (Fig. 1). Here, amyloid refers to aggregates with a cross-β-sheet architecture and long, fibrillar morphology, regardless of the constituent protein or peptide, and the terms amyloid and amyloid fibril are used interchangeably. With Aβ, we denote peptides derived from the amyloid precursor protein (APP) through sequential cleavage by β- and γ-secretases, and we use the term amyloidogenic to describe the capacity of peptides or proteins to misfold and assemble into amyloid fibrils. Clarifying this terminology helps understand the mechanistic overlaps that underlie the disease-disease interactions described throughout this review.

While Aβ is one of the most extensively studied amyloid-forming proteins in the context of AD, other peptides and proteins, including tau, α-synuclein (αSyn), TAR DNA-binding protein 43 (TDP-43), amylin, and even viral peptides, form amyloid fibrils [33–41]. It has been proposed that the ability to form amyloid fibrils is a general feature of polypeptide chains, and any protein, including those that are natively unfolded, can convert into amyloid fibrils under suitable conditions [42–44]. Beyond NDDs, amyloid formation is also implicated in a broad range of systemic disorders such as light chain amyloidosis and transthyretin amyloidosis, further underscoring its pathogenic relevance [45, 46]. By understanding diseases as interconnected rather than isolated entities, we hope to contribute to a paradigm shift in how diagnosis, research, and treatment can be approached in the future.

## Molecular and cellular interactions between diseases

### Viral infections and NDDs

It is increasingly recognized that pathogens can cause a wide variety of human diseases. A key example is the discovery that *Helicobacter pylori* infection leads to peptic ulcers [47]. Those results challenged the longstanding belief that ulcers are caused by stress or spicy foods and led to the development of antibiotic-based treatments. This paradigm shifting has driven further investigations into the role of pathogens in other complex diseases [48]. In this section, we present current evidence linking various viral infections, including those caused by coronavirus and herpesvirus, to the onset of neurological manifestations and the progression of NDDs [8, 49, 50]. Viral infections may activate microglia and astrocytes, prompting the release of pro-inflammatory cytokines and chemokines, which can lead to neuronal death and disrupt synaptic function [51, 52]. Viral infections can also stimulate the production of reactive oxygen species (ROS), inducing oxidative stress and further neuronal damage [53, 54]. These inflammatory processes play a pivotal role in virus-induced neurodegeneration by exacerbating neuronal damage and dysfunction.

#### SARS-CoV-2

The COVID-19 pandemic sparked a profound global health crisis that impacted millions of people worldwide [55]. While respiratory symptoms predominate, there have been indications that SARS-CoV-2, the virus causing COVID-19, can trigger neurological manifestations [56–59]. As many as 50% of COVID-19 patients were reported to experience neurological symptoms, including headache, dizziness, altered mental status, and anosmia [56, 57]. Severe cases may entail seizures, stroke, or encephalitis. Similarly, a UK-wide surveillance study found that 62% of COVID-19 patients with neurological symptoms suffered cerebrovascular events, 31% exhibited altered mental status, and 23% presented peripheral nervous system disorders [57]. Several investigations have suggested a potential association between COVID-19 and NDDs. For instance, viral particles of SARS-CoV-2 were identified in the brain tissues of afflicted individuals [59]. Furthermore, a study conducted in the Chicago area examined the frequency and severity of neurologic manifestations in patients hospitalized with COVID-19. It was found that 82% of 509 COVID-19 patients exhibited neurological manifestations at some point during their illness, with 32% experiencing encephalopathy-associated morbidity [58]. By contrast, other studies reported much lower encephalopathy rates, with 5.7% in a Philippine nationwide study of 10,881 hospitalized patients and 8.7% in the TriNetX COVID-19 Research Network database of 12,601 hospitalized patients [60, 61]. Such discrepancies may be due to the differences in study design, sample size and demographic characteristics, ICU or special unit admission, and inclusion criteria [60]. Collectively, however, these findings suggest the possibility that SARS-CoV-2 may be able to breach the blood–brain barrier (BBB) and cause damage to the brain.

**Fig. 2:**
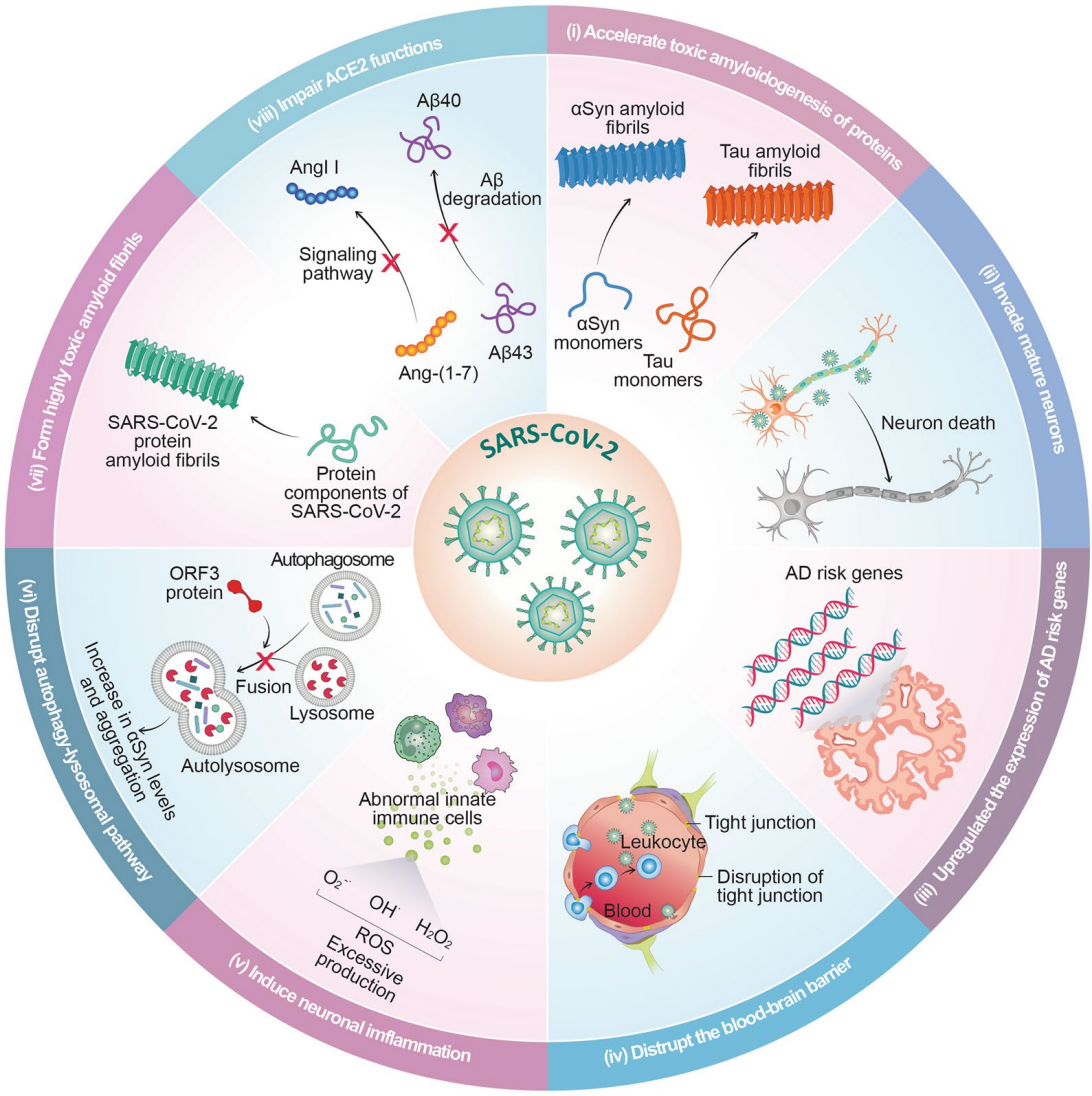
Possible mechanisms by which SARS-CoV-2 may trigger neuropathological processes. The possible mechanisms include: (i) accelerated aggregation of proteins, including αSyn and tau, by SARS-CoV-2 [62–64], (ii) neuronal death resulting from the binding of SARS-CoV-2 to and entry into neurons [8, 65], (iii) upregulation of AD risk genes by SARS-CoV-2 [19], (iv) disruption of the BBB by SARS-CoV-2 [8], (v) induction of innate immune responses by SARS-CoV-2, leading to neuronal inflammation and excessive ROS production [54, 64], (vi) impairment of autophagy-lysosomal pathway by SARS-CoV-2 [79, 80], (vii) formation of amyloid fibrils from protein components of SARS-CoV-2 [38–41], and (viii) dysregulation of ACE2 functions by SARS-CoV-2 [86]

Investigations into the potential mechanisms underlying the neuropathy induced by SARS-CoV-2 have also revealed several key findings (Fig. 2). SARS-CoV-2 may accelerate the progression of tauopathies and synucleinopathies by promoting the amyloidogenesis of αSyn and tau [62–64] and by infiltrating neurons [8, 65]. SARS-CoV-2 also increases the expression of genes potentially linked to AD risk, such as *IL-18*, which is involved in neuroinflammation and amyloid processing, and *KLF4*, which plays a role in regulating neuronal apoptosis [19]. However, the association of these changes of gene expression with the development of neuropathology remains unclear and requires further investigation. Additionally, several studies have demonstrated that SARS-CoV-2 disrupts the integrity of the BBB, leading to increased permeability [66–69]. Components of the SARS-CoV-2, including the spike proteins and the receptor-binding domain, have been shown to directly interact with brain endothelial cells, contributing to BBB disruption [69]. Moreover, SARS-CoV-2 infection also reduces the expression of key BBB tight junction proteins, such as claudin-5 and occludin [67].

SARS-CoV-2 infection can trigger a systemic inflammatory response, characterized by significantly elevated levels of proinflammatory cytokines, such as IL-6, IL-1β, and TNF-α [70–73]. These cytokines can cross the BBB and contribute to sustained dysfunction of microglia, a central feature of COVID-19-related neuroinflammation [74]. In addition to cytokine exposure, direct interactions with viral particles further contribute to microglial impairment [75]. Recent evidence indicates that microglial dysfunction is closely correlated with the levels of viral load and the IL-1- and IL-6-related inflammation [76]. Notably, SARS-CoV-2 also promotes NLRP3 inflammasome activation in microglia through NF-κB signalling and angiotensin-converting enzyme (ACE) 2, providing a mechanistic link between viral infection and neuroinflammation [77]. Moreover, SARS-CoV-2 also enhances the αSyn amyloid fibril-mediated activation of NLRP3 inflammasome by priming human monocyte-derived microglia via the spike protein, highlighting its potential to intensify neuroinflammatory responses relevant to PD [77]. This cascade exacerbates the production of ROS and the release of neurotoxic mediators, ultimately leading to synaptic and neuronal damage [54, 78].

Open reading frame (ORF) 3 is the largest accessory protein of SARS-CoV-2. One of its notable effects is the disruption of autophagic flux, likely by inhibiting autophagosome-lysosome fusion. This leads to the accumulation of autophagosomes and impaired degradation of intracellular substrates [79, 80]. When broadly expressed in the brains of mice, ORF3 induces dysfunction of the autophagy-lysosomal pathway, resulting in a significant increase in αSyn levels in brainstem neurons, which may promote the aggregation of αSyn and contribute to neuropathogenesis [79].

Several protein components of SARS-CoV-2, including the spike protein, a peptide from the NSP6 protein, nucleocapsid protein, and the ORF6, ORF10 and NSP11 proteins, have been found to possess amyloidogenic properties [38–41]. Their amyloid formation can induce significant toxicity to neuronal cells, suggesting a potential link between COVID-19-associated dementia and the amyloid state of SARS-CoV-2 [39, 40]. Moreover, through cross-seeding, these amyloids might catalyse the deposition of other proteins, such as Aβ, potentially exacerbating pre-existing dementia conditions, including AD [41].

ACE2 serves as the primary cellular receptor for SARS-CoV-2, facilitating viral entry into human cells [81]. Beyond its role in viral entry, ACE2 protects the nervous system and regulates inflammation [82]. ACE2 acts in concert with ACE to convert aggregation-prone Aβ43 into less aggregation-prone Aβ40, thereby mitigating Aβ aggregation [83]. Moreover, ACE2 induces the conversion of angiotensin (Ang) II into Ang-(1-7), which activates the Mas receptor (MasR). This ACE2/Ang-(1-7)/MasR signalling pathway promotes neuronal survival and reduces neuroinflammation [84]. A post-mortem study of AD brains revealed that the ACE2 enzymatic activity was significantly decreased with increased Aβ accumulation and higher phosphorylated tau load, suggesting a critical role of ACE2 in maintaining brain health [85]. Upon SARS-CoV-2 infection, the spike protein of the virus binds tightly to ACE2, leading to internalization of the virus–ACE2 complex and often causing the shedding of the ectodomain of ACE2 [86]. As a result, ACE2 activity and function decrease, leading to the impairment of ACE2-mediated Aβ cleavage and anti-inflammatory signalling. These findings highlight that individuals with AD, who already exhibit reduced ACE2 activity, may experience exacerbated AD pathology following SARS-CoV-2 infection. In addition, a recent study reported elevated levels of soluble ACE2 in the brains of individuals with AD [87]. While increased soluble ACE2 may provide additional viral binding sites, it remains unclear whether this directly increases the risk of SARS-CoV-2 entering brain cells and exacerbating neurological symptoms. Further investigation is necessary to clarify the implications of altered ACE2 levels in the context of COVID-19 and AD.

Taken together, these studies suggest the possibility that COVID-19 may trigger diverse neurological manifestations, some of which may lead to severe neurological conditions, and potentially accelerate the progression of pre-existing NDDs [19, 20, 38, 40, 64, 86]. Further research is needed to elucidate the mechanisms responsible for COVID-19-induced neurological damage and its lasting impact on the brain.

#### Human immunodeficiency virus (HIV)

First identified in the 1980s, HIV leads to acquired immunodeficiency syndrome (AIDS) and has resulted in over 25 million deaths, with a prevalence in sub-Saharan Africa [88, 89]. While the viral concentration in seminal fluid is important, certain components of semen also influence the viral infectivity. One such component is semen-derived enhancer of viral infection (SEVI), an amyloid fibril formed from fragments of prostatic acid phosphatase (PAP) [90]. SEVI occurs naturally in semen, regardless of HIV status, and has been shown to significantly enhance HIV infectivity [90, 91]. This enhancement occurs through facilitating membrane fusion between the host cell and the HIV virion. [88]. This observation underscores the potential of amyloidogenesis to amplify infectious diseases, suggesting a link between protein misfolding diseases and infectious diseases.

The complex nature of the interplay between proteins associated with different diseases is illustrated by the interaction between SEVI and Aβ. Studies using various methodologies, including spectroscopies, cell assays, and transgenic worm models, found that SEVI could interact with Aβ monomers and oligomers, effectively inhibiting amyloid fibril formation [92]. Additionally, SEVI has demonstrated the capability to disaggregate preformed Aβ amyloid fibrils into smaller amorphous aggregates [92]. The interactions between SEVI and Aβ protect against Aβ-induced toxicity in neurons and transgenic worms, resulting in increased cell viability, reduced cytotoxicity, diminished worm paralysis, and an extended lifespan in the worms [92].

While these findings suggest that SEVI may inhibit Aβ aggregation and toxicity in experimental models, its relevance to the pathology of AD remains uncertain. Clinical data have shown that women over the age of 65 are approximately 10% more likely to develop AD than men of the same age group [92, 93]. Although it has been proposed that SEVI might provide a protective effect against AD in males, sex-related differences in AD risk are more commonly attributed to sex difference in the association of apolipoprotein E (*APOE*) ε4 with AD [94–96], sex-specific variations in tau pathology [97–100], and sex hormones [101]. Additionally, some studies have reported that precursors of peripheral amyloid fibrils, such as serum amyloid A and prion proteins, can cross the BBB and accumulate in the brain [102, 103]. The endogenous expression of PAP, the precursor of SEVI, has also been detected in the brain [104]. However, there is currently no direct evidence demonstrating the presence of SEVI in the central nervous system (CNS). Further research is needed to determine whether SEVI can enter the brain and to fully elucidate the pathological relationships and mechanisms underlying the relationship between Aβ-AD and SEVI-HIV/AIDS.

#### Herpes simplex virus type 1 (HSV-1)

**Fig. 3:**
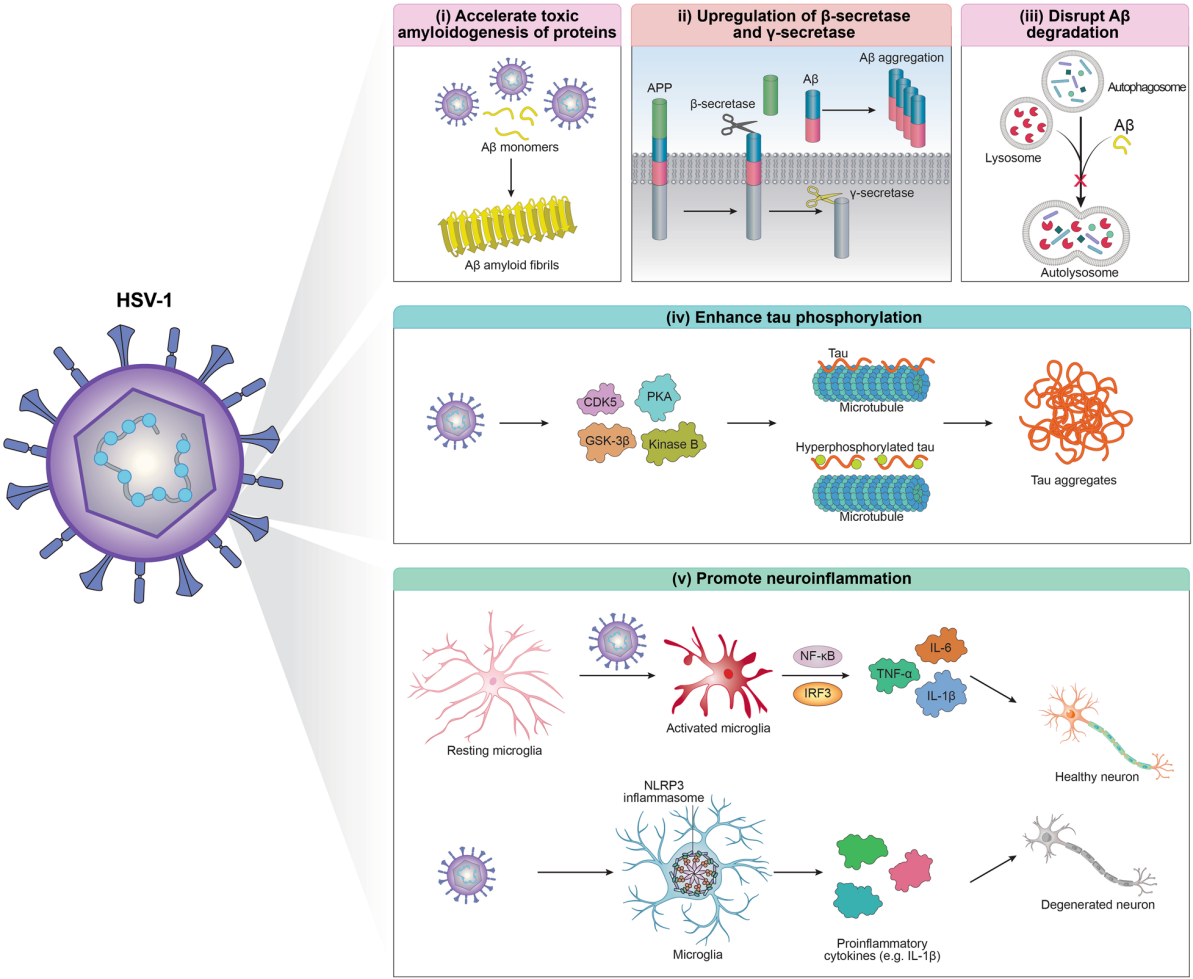
Possible mechanisms of neuronal toxicity in HSV-1 infections, including: (i) accelerated formation of Aβ amyloids by HSV-1 [49], (ii) increased production of Aβ by HSV-1 [110, 111, 113], (iii) impaired clearance of Aβ by HSV-1 [115], (iv) enhanced tau phosphorylation by HSV-1 [116, 117], and (v) promotion of neuroinflammation by HSV-1 [120, 126]

Previous studies have suggested a potential association between members of the Herpesviridae family and late-onset dementia in AD [105, 106]. HSV-1, which infects around 80% of people over 60, has been suggested to increase AD risk in individuals carrying the *APOE* ɛ4 allele [107, 108]. Earlier research has identified an elevated presence of Herpesviridae in the brains of AD patients and has demonstrated that herpes viruses promote the aggregation of Aβ (Fig. 3) [49, 109]. Moreover, in vitro experiments have demonstrated that HSV-1 may accelerate Aβ amyloid fibrillation by enhancing the nucleation step [110]. One proposed mechanism involves Aβ binding to envelope glycoproteins on the surface of HSV-1 particles [111], thereby increasing the local peptide concentration and favouring aggregation. Biological membrane mimetics and nanoparticles have also been observed to boost amyloid fibrillation through surface-induced enhancement of local protein concentration [34, 35, 112]. In addition to directly influencing the aggregation kinetics, HSV-1 may promote Aβ accumulation by adjusting its production and clearance. A previous study has reported significantly higher levels of Aβ42 in HSV-1-infected neuroblastoma cells over mock-infected controls [113], which may result from HSV-1-induced upregulation of β-secretase and nicastrin, a key component of the γ-secretase complex (Fig. 3). Both β- and γ-secretases are essential for processing APP into Aβ [114]. Meanwhile, HSV-1 infection causes an intense accumulation of autophagic vesicles and impairs the fusion between autophagosomes and lysosomes in infected human neuroblastoma cells (Fig. 3) [115]. This dysfunction in the late stages of autophagy consequently disrupts Aβ clearance.

Beyond Aβ-related effects, HSV-1 also contributes to the development of AD through other mechanisms, including tau hyperphosphorylation and neuroinflammation. HSV-1-infected neuroblastoma cells exhibit elevated levels of hyperphosphorylated tau [116, 117], possibly via increased activity of cyclin-dependent kinase 5, glycogen synthase kinase-3β (GSK-3β), protein kinase A (PKA), and kinase B (Fig. 3) [116, 118, 119]. Excessive phosphorylation of tau causes its detachment from microtubules and aggregation into amyloid fibrils, which accumulate as neurofibrillary tangles (NFTs). On the other hand, HSV-1 has also been implicated in promoting neuroinflammation by activating microglia and triggering the NF-κB and IRF3 signalling pathways [120, 121], leading to the release of pro-inflammatory cytokines, such as IL-6, TNF-α, and IL-1β [122]. Of particular interest, recent studies have linked HSV-1 infection to the activation of NLRP3 inflammasomes, which can impair Aβ clearance, trigger the release of pro-inflammatory cytokines, and promote tau hyperphosphorylation and aggregation (Fig. 3) [123–125]. In 5 × FAD mice, HSV-1 has been reported to activate NLRP3 inflammasomes, resulting in increased Aβ deposition and cognitive deficits [126]. These findings suggest that the NLRP3 inflammasome activation may play a key role in linking HSV-1 infection to Aβ accumulation in AD.

In addition to AD, HSV-1 is also associated with an increased risk of senile dementia [127]. Moreover, the incidence of HSV-1 infection is significantly higher in patients with idiopathic PD compared to healthy controls [128]. These studies highlight the potential role of HSV-1 in various types of NDDs. Despite this compelling experimental evidence, a definitive causal link between HSV-1 and NDDs remains unclear. Given the high prevalence of HSV-1 in older population, the association between HSV-1 and AD may be affected by age-related immune changes or other biological factors. Further investigations are warranted to investigate these relationships across diverse populations and individuals with existing dementia, while considering other factors such as bacterial infections. Furthermore, compared to HSV-1, HSV-2 has received much less attention, although some studies have reported a link between HSV-2 and AD and amyotrophic lateral sclerosis (ALS), the most common motor neuron disorder [129, 130]. Detailed investigations are needed to further explore the relationship between HSV-2 and NDDs.

#### Epstein–Barr virus (EBV)

Previous studies have suggested a possible link between EBV and AD [131, 132]. In a study involving 93 AD patients and 164 healthy individuals, EBV DNA was detected in the blood of about 45% of AD patients, compared to 31% of the control group [131]. This association is further demonstrated in a larger Mendelian randomization study, which showed a significant link between mononucleosis, a pathology caused by EBV, and an increased risk of AD [132].

To better understand the implication of EBV in AD, extensive in vitro and in vivo studies have been conducted. EBV increases the synthesis of APP, possibly through the elevation of NF-κB levels. This upregulation of APP may, in turn, enhance the generation of Aβ peptides [133]. Moreover, EBV activates inflammasomes and stimulates the release of inflammatory molecules, such as IL-1β, IL-8, and TNF-α [133–135]. These pro-inflammatory factors worsen neuroinflammation and contribute to both Aβ accumulation and tau hyperphosphorylation [136]. EBV infection may also interfere with Aβ clearance by disrupting the function of APOE, particularly the APOE E3 isoform. The C-terminal region of APOE E3 has been shown to interact with EBV proteins like Epstein–Barr nuclear antigen 1 (EBNA1) and BamHI Z fragment leftward open reading frame 1 (BZLF1), impairing its ability to clear Aβ [137]. Collectively, these results highlight multiple pathways through which EBV may promote AD-related pathology.

Interestingly, recent studies on EBV protein aggregation provide novel insights into how EBV infection may be linked to Aβ aggregation [133, 135, 138]. Using computational screening, researchers identified a 12-amino-acid peptide (146SYKHVFLSAFVY157) from EBV glycoprotein M (EBV-gM) that displays aggregation and hydrophobicity profiles similar to Aβ42 [138]. This EBV-gM_146–157_ fragment can aggregate into spheroid aggregates in vitro. Additional experiments showed that the presence of this peptide promotes the aggregation of Aβ42 [133]. Although EBV-gM_146–157_ lies between two predicted 20S proteasomal cleavage sites, it has not yet been detected in vivo. Further research is needed to determine whether the aggregation of EBV proteins plays a role in AD.

In addition to its potential involvement in AD, mounting evidence suggests that EBV infection may target B cells and contribute to the onset of multiple sclerosis (MS) [139, 140]. A dose-dependent relationship has been observed between MS risk and levels of EBV-specific antibodies, particularly EBNA-1 IgG titers [141]. One proposed mechanism involves molecular mimicry between EBV nuclear antigen 1 and glial cell adhesion molecule, a protein in the brain and spinal cord, which may generate autoimmune T cells or B cells that mistakenly target myelin [142, 143]. EBV infection also generates a lifelong reservoir of EBV-infected B cells, promoting chronic inflammation in the CNS [140]. Further investigation is needed to elucidate the specificity and determinants of the immune response to EBV, however. Conflicting results regarding the presence and quantity of EBV DNA in blood, cerebrospinal fluid, or the brain have been reported across studies [144–146]. While dysfunction of CD8^+^ cytotoxic T cells, responsible for immune surveillance, is observed in MS, consistent evidence of EBV reactivation or increased viral DNA load in cellular compartments is lacking [147]. More sensitive quantitative assays may help resolve these discrepancies. Additional research is also warranted to explore the role of CD4 cells, considering additive interactions between markers of EBV infection and HLA class II genes involved in antigen presentation. Although elevated anti-EBNA IgG titers are consistently observed, evidence of increased EBV genome copies or serological reactivation remains inconsistent [140]. Achieving greater consistency in findings through larger sample sizes, well-characterized MS and control populations, and advanced laboratory techniques is essential to advance our understanding of the EBV–MS connection.

### Cancer and NDDs

Recent research has unveiled a nuanced relationship between cancer and NDDs, notably AD [9, 18, 148–150]. Most epidemiological evidence suggests an inverse association between these conditions, with cancer survivors exhibiting a 20%–50% lower risk of developing AD and PD, and patients with NDDs demonstrating a substantially lower incidence of cancer [18, 151]. Despite this inverse association, however, the diseases also share common mechanisms and risk factors, indicating a potential link between their pathological progression [9]. This prompts further investigation to better understand the intricate connection between cancer and neurodegeneration.

#### Cancer and AD

There is limited knowledge on the connection between cancer and AD. Autopsy studies initially revealed that AD patients had fewer incidental cases of cancer compared to those without AD [152]. Two independent studies demonstrated an inverse association between AD and the occurrence of lung cancer [151, 153]. Moreover, a subsequent transcriptomic meta-analysis indicated that this relationship is linked to the deregulation of several genes in opposite directions in AD and lung cancer [154].

Notably, individuals diagnosed with AD have a significantly lower risk of prior cancer compared to age-matched controls, while those with vascular dementia have a higher risk [18, 155]. Cohort studies in recent years have validated these initial findings [156–159]. People with AD exhibit a reduced risk of developing incident cancer, and those with prevalent cancer have a lower risk of probable AD [157, 158]. The inverse relationship between cancer and AD has been consistently observed in various cohort studies, indicating that the decreased risk is not solely attributable to mortality differences between groups [151]. The findings also suggest that the reduced risk of AD in cancer survivors is not due to a general reduction in late-onset diseases. Similarly, the lower risk of cancer diagnosis in AD patients is not explained by under-diagnosis or under-reporting associated with cognitive impairment, as it is not observed in individuals with vascular dementia [157].

At the molecular level, key signalling pathways are often regulated in opposite directions in AD and cancer. For instance, the PI3K/Akt/mTOR axis, which is commonly upregulated in cancer to promote survival and growth, is frequently impaired in AD, contributing to neurodegeneration through reduced synaptic plasticity and increased tau phosphorylation [14, 160, 161]. Notably, sex hormones, such as oestrogens and androgens, have been reported to drive several cancers through activating the PI3k/Akt pathway [162]. While PI3k/Akt overactivation promotes oncogenesis, it also inhibits GSK3β, thereby preventing tau hyperphosphorylation, amyloid formation, and subsequent deposition into NFTs. Supporting this neuroprotective role, experimental studies have revealed that oestrogen depletion increases age-related accumulation of phosphorylated tau in the hippocampi of rats [163], whereas oestrogen treatment attenuates tau hyperphosphorylation in the neuroblasts of mice [164]. Similarly, androgen has been reported to reduce tau phosphorylation in an androgen receptor-dependent manner [165].

Tumour suppressor p53 also exhibits dual behaviour across these diseases [9, 166, 167]. In cancer, loss-of-function mutations in p53 drive cancer by impairing cell cycle arrest and apoptosis. Most of these mutations are missense mutations that occur within the central DNA-binding domain and are commonly observed across a wide range of human malignancies, including breast cancer and gastric cancer [168–170]. In contrast, in AD, p53 is frequently hyperactivated in neurons and contributes to apoptosis and synaptic dysfunction. Previous studies in the brains of AD patients and mouse models have shown that p53 is frequently upregulated with enhanced activity, promoting neurodegeneration [171–174]. Notably, the accumulation of Aβ appears to be a trigger for the enhanced p53-mediated apoptosis. In AD transgenic mice and cultured hippocampal neurons, Aβ exposure increases the expression of PUMA (p53-upregulated modulator apoptosis), a pro-apoptotic protein [175], and induces p53 phosphorylation at serine 15, which enhances p53 pro-apoptotic transcriptional activity [176]. These findings suggest that Aβ accumulation may exacerbate neuronal vulnerability in AD by amplifying p53-driven apoptotic pathways, supporting the notion that the hyperactive p53-mediated apoptosis may partially underlie the inverse relationship between AD and cancer.

Chronic hypoxia is a critical feature observed in both AD and cancer, and it has been suggested to contribute to their inverse relationship through multiple molecular mechanisms, including the differential regulation of hypoxia-inducible factor-1α (HIF-1α), the Warburg and reverse-Warburg metabolic effects, lactate-mediated alterations in intracellular pH, and VDAC1 (voltage-dependent anion channel 1)-mediated apoptotic and anti-apoptotic signalling [177]. For instance, HIF-1α is often upregulated in breast and lung cancer [178, 179]. Previous studies have suggested that HIF-1 mediates the increased expression of glucose transport (GLUT) 1 and GLUT3, which promotes glucose uptake to support the proliferation and division of cancer cells [180–182]. Interestingly, the levels of GLUT1 and GLUT3 have been reported to inversely correlate with tau hyperphosphorylation and NFT intensity in the brain [183]. Therefore, elevated GLUT1 and GLUT3 levels in cancer may help reduce tau aggregation. Indeed, HIF-1 is downregulated in AD [183]. Collectively, these findings suggest that HIF-1α serves as a key mediator at the crossroad of cancer and AD.

*APOE* genotype has received increasing attention for its potential role in mediating the link between AD and cancer [184, 185]. The three common alleles of *APOE* gene, ɛ2, ɛ3, and ɛ4, encode the corresponding protein isoforms ApoE2, ApoE3, and ApoE4, respectively [186]. Substantial evidence shows that *APOE* ɛ4 is the strongest genetic risk factor for AD when compared to ɛ2 and ɛ3 [187–189]*.* One possible explanation is that ApoE4 is less effective at promoting Aβ clearance [190, 191]. On the other hand, *APOE* is also involved in cancer. The latest evidence suggests that APOE expression is positively associated with anti-tumour immune signatures and prevalent in early-stage tumours [192]. Interestingly, among APOE genotypes, *APOE* ɛ4 may possess greater anti-tumour effects. As shown in PC12 cell models, ApoE4 more strongly inhibits the canonical Wnt signalling, a key pathway in cell proliferation and survival, than the other isoforms [193]. Moreover, mice carrying human *APOE* ɛ4 show slower melanoma growth and spread compared to those with *APOE* ɛ2, likely due to a stronger anti-tumour immune response [194]. Nevertheless, the role of *APOE* in cancer is complex and appears to be context-dependent [184]. Further research is needed to uncover how *APOE* variants affect the development of different cancers, which will provide further insights into their role in the interplay between cancer and AD.

#### Cancer and PD

PD is a movement disorder resulting from the degeneration of dopamine-producing neurons in the substantia nigra. PD consistently demonstrates a lower risk for most cancers, including both smoking-related and non-smoking-related types [195–199]. Large-scale epidemiological studies utilizing patient registries have consistently reported a reduced incidence of overall cancer in PD patients [18, 200]. This reduction in cancer risk has also been observed in studies involving US physicians with PD [201, 202]. A meta-analysis of multiple studies further confirmed the decreased risk of overall cancer, smoking-related cancer, and non-smoking-related cancer in PD patients [195]. Moreover, several longitudinal and cohort studies based on data from the Korean National Health Insurance database have unveiled a substantial inverse relationship between PD and cancer [199, 203]. Notably, PD patients face an increased risk of specific cancers, such as malignant melanoma [204]. This heightened risk may be attributed to shared characteristics between pigmented cells in the brain and skin [204]. Nonetheless, the precise reasons behind the inverse association between PD and cancer risk remain incompletely understood.

Recent molecular studies suggest that shared biological pathways that are dysregulated in opposite directions may explain the inverse association between PD and cancer. Mutations or loss-of-function in certain PD-associated genes, such as *PINK1*, *PARK2*, and *DJ-1*, impairs mitochondrial respiration, disrupts mitophagy, and leads to the accumulation of damaged mitochondria, resulting in oxidative stress and neuronal death [205–209]. In contrast, these same alterations can enhance mitochondrial turnover and support metabolic reprogramming in proliferating cells, potentially reducing cancer risk by limiting uncontrolled cell growth and survival [205, 210]. For example, mutations in *DJ-1* cause rare familial PD, likely by impairing cellular response to oxidative stress [211]. One critical function of DJ-1 is stabilizing the nuclear factor erythroid 2-related factor (Nrf2) by preventing its degradation, thereby promoting the expression of detoxification enzymes and ROS defence mechanisms that protect neurons from oxidative injury [212]. In PD, DJ-1 deficiency destabilizes Nrf2, weakening antioxidant defences and rendering neurons more susceptible to oxidative damage and apoptosis. Nonetheless, DJ-1 is frequently upregulated in many malignancies, such as lung, breast, colorectal, liver, and melanoma, where it promotes tumour progression and metastasis by activating Nrf2- and Akt-mediated pro-survival signalling pathways [199, 212–214]. Loss of DJ-1 reduces inhibition of the tumour suppressor PTEN, thereby damping PI3K/Akt signalling and attenuating cell proliferation [215, 216]. Thus, individuals with DJ-1 deficiency may have a lower propensity for cancers driven by DJ-1-mediated pathways. Overall, DJ-1 enhances cell survival and growth pathways that benefit cancer progression but may contribute to neuronal loss in PD when dysregulated.

αSyn plays diverse physiological roles, particularly in neurons where it maintains neurotransmitter release by regulating synaptic vesicle pools and facilitating SNARE complex assembly [217]. In PD, misfolded αSyn forms amyloid fibrils that deposit as Lewy bodies, leading to synaptic dysfunction, oxidative stress, and activation of apoptotic pathways [218–220]. Recent studies have also highlighted the role of αSyn in cancer, where it exerts context-dependent effects. For example, αSyn is expressed in various malignancies, including ovarian cancer, breast cancer, and melanoma, where its elevated levels have been associated with tumour-promoting functions [221–224]. In MG63 osteosarcoma cells, αSyn promotes differentiation toward an osteoblastic phenotype by suppressing proteasome activity and protein kinase C (PKC) signalling pathway, while enhancing lysosomal function [225]. Additionally, αSyn modulates key oncogenic pathways. In meningioma, αSyn upregulation alters the phosphorylation status of Akt/mTOR components, thereby affecting cell proliferation, apoptosis, migration, and invasion [224]. Nonetheless, several studies also have identified tumour-suppressive effects of αSyn. For instance, in lung adenocarcinoma, αSyn expression is suppressed via promoter hypermethylation, and its overexpression inhibits the PI3K/Akt/mTOR signalling pathway, leading to reduced tumour cell proliferation [226]. Similarly, a recent study demonstrated that exosome-delivered αSyn inhibits proliferation, migration, and invasion of hepatocellular carcinoma cells; this anti-tumour effect is further enhanced by the presence of integrin αVβ5 in exosomes. In vivo experiments in rat models confirmed that exosomal αSyn effectively suppresses liver cancer progression [227].

As discussed above, diverse molecular mediators (e.g., Parkin, αSyn) have been shown to play differing roles in cancer and PD [9]. In addition to these biological factors, one possible environmental contributor to the reduced cancer incidence observed in PD patients is the lower prevalence of smoking in this population, which may partially account for the inverse association. Additionally, the elevated mortality rate among individuals with PD could reduce the likelihood of cancer development simply due to shortened lifespan [18]. Given the complexity of the association between PD and cancer, further research is necessary to elucidate the underlying mechanisms. Understanding these complexities could have significant implications for both PD management and cancer prevention strategies.

#### Cancer and Huntington’s disease (HD)

Epidemiological studies investigating the relationship between cancer and HD remain relatively limited [228–230]. Two studies utilizing data from the Danish National Cancer Registry and Swedish Cancer Registry found an inverse association between HD and cancer risk, with lower incidence rates observed in HD patients compared to the general population [228, 229]. Similarly, a recent study with 6540 subjects in the European Huntington’s Disease Network REGISTRY reported a lower incidence of cancer in individuals with HD [230].

One key mechanism responsible for this inverse association is the enhancement of apoptosis [231, 232]. HD is caused by the expression of mutant huntingtin (mHTT), which undergoes misfolding and aggregation, disrupts cellular homeostasis, and ultimately leads to progressive neurodegeneration [233]. However, mHTT may also play a protective role against cancer via promoting apoptosis. Previous studies have shown that the presence of mHTT in both neuronal and peripheral cells increases p53 activity and lowers the apoptotic threshold, thereby promoting caspase-6 activation and apoptotic cell death [232]. Additionally, mHTT has been reported to reduce the efficiency of DNA repair by disrupting poly (ADP-ribose) polymerase-mediated signalling [234], which is more likely to induce apoptotic cell death, rather than driving malignant transformation.

Another proposed mechanism involves the generation of small interfering RNAs (siRNAs) from the mutated huntingtin gene in individuals with HD compared to the general population [235]. These siRNAs have demonstrated a remarkable ability to selectively target and eliminate cancer cells while sparing healthy cells, holding significant promise for potential therapeutic applications in cancer treatment. Furthermore, a recent hypothesis suggests that the upregulation of chaperone-mediated autophagy (CMA) may also contribute to the reduced cancer risk in HD [236]. mHTT aggregation has been suggested to enhance CMA, a selective degradation pathway for misfolded proteins [237–239]. This enhancement is associated with increased levels of key CMA components, including the cytosolic chaperone HSC70 and the lysosome-associated membrane protein type 2A, as observed in HD cells [239]. Notably, CMA upregulation regulates oncogenic proteins, such as MDM2 (mouse double minute 2) and MYC, thereby contributing to tumour suppression [240–242].

#### Cancer and ALS

ALS is a neurodegenerative disorder that primarily affects motor neurons. A study in England found no significant association between ALS and cancer when comparing cancer risk before and after ALS diagnosis [243]. An increased risk of Hodgkin’s lymphoma and brain tumours, however, was noted around the time of ALS diagnosis [244]. Another study using a cancer registry found no overall association between ALS and cancer mortality, although specific associations with prostate cancer, melanoma, tongue cancer, and leukaemia were noted [245]. In support of site-specific changes in cancer risks among ALS patients, a substantial observational longitudinal study was published [246]. Drawing from data in the Utah Population Database, this study revealed that individuals with ALS exhibited a decreased risk of developing lung cancer while experiencing an elevated risk for salivary and testicular cancers [246]. These findings underscore the complexity of the association between ALS and cancer, which will require further investigations to be clarified.

Emerging evidence indicates that RNA-binding proteins (RBPs) play a pivotal role in the molecular link between ALS and cancer [247]. TDP-43 is a principal pathogenic RBP in ALS, where it aggregates aberrantly and forms cytoplasmic inclusions. This process leads to disrupted cellular functions and neurodegeneration [248]. Interestingly, TDP-43 also exhibits oncogenic properties in several cancers when it remains functionally intact [249–251]. In lung cancer, TDP-43 regulates the expression of specific microRNAs and interact with mature microRNAs, thereby promoting tumour progression [249]. Another study reported that knockdown of TDP-43 downregulates the long non-coding RNA *MALAT1*, resulting in reduced cancer cell proliferation and migration [250]. In melanoma, TDP-43 has been observed to be overexpressed. TDP-43 knockdown inhibits cancer cell proliferation and metastasis by suppressing GLUT4 and reducing glucose uptake [251]. Beyond TDP-43, other ALS-associated RBPs, such as FUS (Fused in Sarcoma) and hnRNPA1 (heterogeneous nuclear ribonucleoprotein A1), have also been suggested to be involved in the link between ALS and cancer [252–254].

#### Toxic impact of amyloidogenic proteins on cancer cells

The toxic effects of amyloidogenic proteins on cancer cells have been investigated to understand the mechanisms underlying the inverse correlation between cancer and NDDs [255, 256]. An analysis with two types of Aβ oligomers was performed on various cancer cell lines, including NB4 (human acute promyelocytic leukaemia), A549 (human lung cancer), and MCF-7 (human breast cancer) [255]. The two oligomer types were: (i) small oligomers (mainly trimers with 42.9% ± 2.0% β-sheet content) prepared using the HFIP protocol, and (ii) large oligomers (mainly pentadecamers with 79.4% ± 9.4% β-sheet content) prepared under HFIP-free conditions. Both types of Aβ oligomers were found to inhibit the growth of all cancer cell lines. Notably, the inhibitory effect varied depending on the type of oligomers and the specific cell line. Aβ oligomers rich in antiparallel β-sheet structural elements exhibited more severe detrimental effects, suggesting that the antiparallel β-sheet structure is a critical determinant of the toxic impact [255]. In A549 and MCF-7 cancer cells, Aβ tended to aggregate in the cell membranes, leading to membrane disruption and decreased cell viability. In NB4 cells, however, Aβ predominantly localizes near the nucleus, displaying distinct and more pronounced effects compared to A549 and MCF-7 cancer cells. Notably, cancer cells appear to be less vulnerable to Aβ42 oligomers than mixed neuronal cultures [255, 257]. In addition to oligomers, the toxicity of Aβ monomers against cancer cells has also been observed. Aβ monomers predominantly inhibit pancreatic cell growth through ROS production [256]. Furthermore, apart from Aβ, amylin and human calcitonin were found to inhibit the proliferation of cancer cells [256]. Further research is needed to clarify the relative sensitivity of cancer cells to amyloidogenic proteins compared to other cell types, particular under physiologically relevant conditions.

It is important to note that the toxic effects of Aβ are generally non-specific and not limited to neurons or cancer cells. Recent studies have shown that Aβ amyloid fibrils can damage brain immune cells, such as macrophages and dendritic cells [258]. Aβ has also been found in peripheral blood, where it may contribute to the pathology of AD by affecting peripheral innate immune cells [259]. Aβ monomers have shown cytotoxic effects on the THP-1 human monocytic leukaemia cell line [260]. Oligomeric Aβ has also been reported to increase ROS in fibroblasts from AD patients, leading to cell death [261]. In addition, current conclusions about the toxicity of Aβ species toward cancer cells are based only on two experimental studies discussed above, and these findings have not been independently confirmed by other groups. Given the non-specific nature of toxicity and the limited number of supporting studies, the overall significance and reliability of this toxic effect of amyloidogenic proteins in the context of cancer and NDDs interactions remain uncertain. Further studies are needed to validate these results, better understand cell-specific vulnerability, and assess whether the toxicity of amyloidogenic proteins could be safely and effectively used in cancer therapy.

#### p53 Aggregation, cancer, and AD

**Fig. 4:**
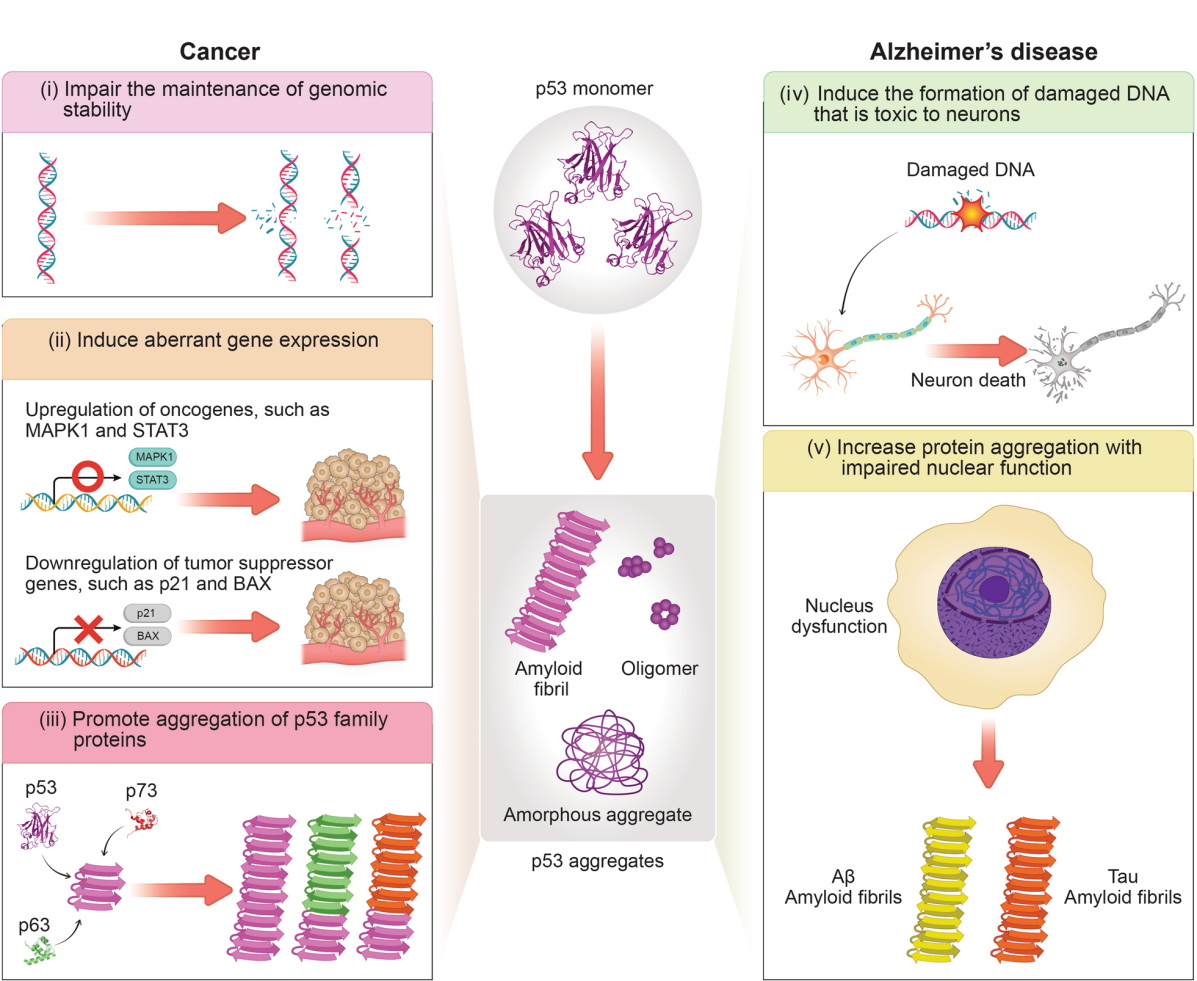
Possible mechanisms by which p53 aggregation may influence cancer and Alzheimer’s disease, including: (i) impairment of genomic stability maintenance [356], (ii) induction of aberrant gene expression [268], and (iii) promotion of p53 family protein aggregation [17, 271] in cancer, as well as (iv) formation of damaged DNA that is toxic to neurons [274] and (v) increased protein aggregation with nuclear dysfunction [275] in Alzheimer’s disease

Soluble p53 monomers can undergo self-assembly, forming insoluble amorphous aggregates or amyloid fibrils under stress conditions (Fig. 4) [262–264]. The presence of p53 aggregates has been observed in various tumours, suggesting a potential role in cancer development [265–267]. P53 aggregates may disrupt normal gene expression by suppressing cell cycle regulators and activating genes that promote cell proliferation [268, 269], thereby contributing to tumorigenesis (Fig. 4). In addition, p53 aggregates can promote the co-aggregation of p63 and p73, further weakening the cellular defence against tumours and increasing the likelihood of tumorigenesis (Fig. 4) [270, 271].

Recent investigations have shed considerable light on the seeding capability of p53 aggregates [17, 272, 273]. Full-length p53 was reported to aggregate into amyloid fibrils, spontaneously forming amyloids under physiological conditions via primary nucleation [273]. Moreover, the aggregation rate was accelerated by the addition of preformed p53 amyloid fibrils, indicating seeded amyloid formation and secondary nucleation [272, 273]. Furthermore, cellular experiments provided evidence that fibrillar seeds of the amyloidogenic region of p53 can be internalized into cells, acting as templates for the aggregation of endogenous p53 [17].

Interactions between p53 and tau, a key factor in AD, have been reported [274, 275]. Tau oligomers were found to interact with p53 in the AD brain, leading to the sequestration and formation of p53 oligomers and amyloid fibrils, ultimately promoting DNA damage (Fig. 4) [274]. Additionally, the aggregation-induced loss-of-function of p53 may enhance protein aggregation and cause cell death [275]. These observations imply that, despite the general inverse association between cancer and AD, the two conditions may share overlapping pathogenic mechanisms under specific conditions. In particular, aberrant protein aggregation involving p53 may represent a convergent pathological mechanism in patients affected by both AD and cancer. Further studies are needed to clarify the interplay between aggregations of p53 and amyloidogenic proteins associated with NDDs. Exploring p53 aggregation and its interactions with other molecular pathways could also provide valuable insights into the pathogenesis of both cancer and NDDs. Additionally, such investigations may uncover novel therapeutic targets for managing and preventing these complex and devastating conditions.

### Diabetes and NDDs

T2D is a chronic metabolic disorder characterized by insulin resistance and impaired glucose regulation [276]. T2D is associated with various forms of cognitive dysfunction, including diabetes-associated cognitive decline, mild cognitive impairment, and dementia, which can occur independently of Aβ accumulation [21]. Epidemiological studies consistently demonstrate a strong link between T2D and AD [277, 278]. The precise molecular mechanism underlying this association remains unclear, however.

Both T2D and AD are characterized as protein misfolding disorders, classified by the aggregation of specific proteins in specific tissues. In AD, Aβ and tau proteins aggregate and accumulate in the brain, while in T2D, aggregates of amylin, also known as islet amyloid polypeptide, are deposited in pancreatic islets [21, 33, 37, 279–286]. In addition to the primary nucleation model of amyloid formation, these protein aggregates follow a secondary nucleation model, where misfolded aggregates act as seeds that promote the misfolding and aggregation of native proteins [287].

**Fig. 5:**
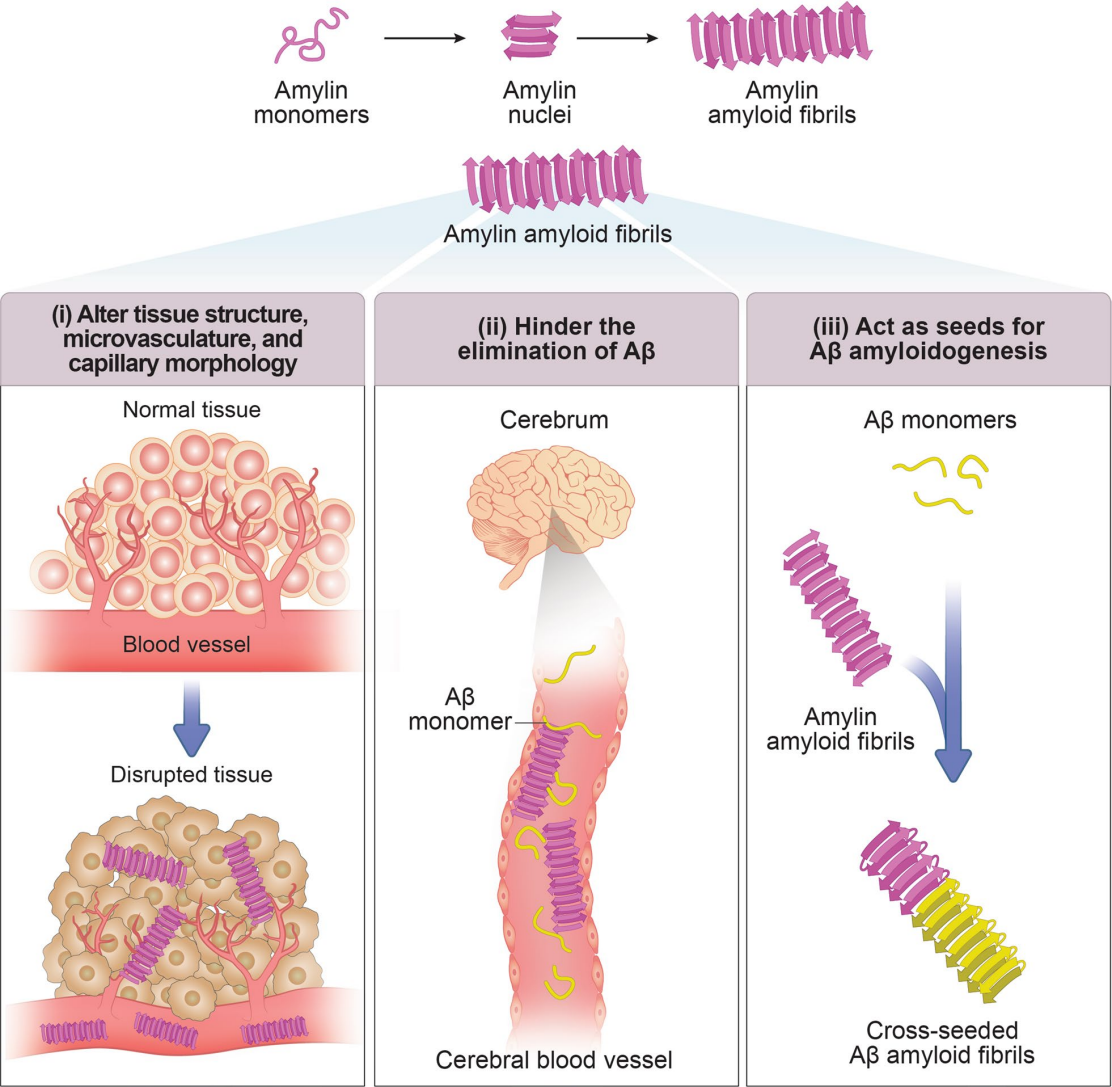
Schematic representation of amylin amyloid-related neurotoxic effects. Among the possible mechanisms, we mention: (i) disruption of tissue structure, microvasculature, and capillary morphology [288], (ii) formation of plaques in conjunction with Aβ, impeding its removal [290], and (iii) cross-seeding of Aβ amyloid formation by amylin amyloid fibrils [15]

Hyperamylinemia, a common pancreatic disorder in obese and insulin-resistant patients [288], triggers the oligomerization and cytotoxicity of amylin, leading to the depletion of β-cell mass and the development of T2D [289]. Studies have revealed the presence of oligomerized amylin in the cerebrovascular system and brain parenchyma of diabetic patients, amylin oligomers and plaques in the temporal lobe grey matter, and extensive amylin deposition in blood vessels and perivascular spaces [288–290]. Amylin deposition was also observed in the blood vessels and brain parenchyma of patients with late-onset AD who did not have clinically apparent diabetes [289]. In some cases, amylin co-deposits with Aβ in the brain [288, 290]. As the brain does not synthesize amylin, the amylin aggregates may enter the brain by crossing the BBB [291, 292]. Amylin accumulation independently leads to amyloid formation and alters tissue structure, microvasculature, and capillary morphology (Fig. 5) [288]. Metabolic disorders and aging contribute to the accumulation of amyloid form of amylin in the cerebrovascular system and grey matter [288]. Furthermore, amyloid formation by amylin in the walls of cerebral blood vessels may hinder the elimination of Aβ, potentially contributing to the etiology of AD (Fig. 5) [290]. Thus, amylin derived from the pancreas accumulates in the brain, forming independent plaques or co-depositing with Aβ to create complex amylin-Aβ plaques [288]. The mechanisms underlying brain amylin accumulation likely involve insulin resistance, hyperamylinemia, and the presence of circulating amylin aggregates, such as oligomers [288–290]. Impaired clearance of proteinaceous residues may also contribute to the accumulation of amylin oligomers [293]. Overall, the accumulation of amylin in aggregated form in the brain, resulting from prediabetic insulin resistance, may contribute to the complex pathology of age-related vascular dementia and NDDs [288].

Misfolded amylin, produced in T2D, enhances the pathology of AD by cross-seeding Aβ, offering a molecular explanation for the association between these conditions [15, 294]. Introduction of amylin seeds accelerates Aβ aggregation in vitro, mimicking the seeding effect, and the resulting amyloid fibrils consist of both peptides (Fig. 5) [15]. Transgenic animals expressing both human proteins exhibit more severe AD-like pathology, compared to AD transgenic mice or AD transgenic animals with type 1 diabetes (T1D) [15]. Notably, amylin was found to co-localize with amyloid plaques in brain parenchymal deposits, indicating a potential direct interaction between these peptides that may exacerbate disease progression [15]. Moreover, injection of pancreatic amylin aggregates into the brains of AD transgenic mice resulted in increased AD pathology and significantly greater memory impairments, relative to untreated animals [15]. These findings serve as proof-of-concept for a disease mechanism involving the interaction of misfolded proteins through cross-seeding events, which may contribute to the acceleration or exacerbation of disease progression.

Although the precise link between T2D and AD remains on debate, imbalances in glucose metabolism have been implicated in the increased risk of AD [295, 296]. This relationship is so significant that some experts have referred to AD, potentially arising from insulin resistance in the brain, as type 3 diabetes [295]. In the case of brain insulin resistance, the clearance of Aβ is significantly disrupted. Insulin resistance can reduce the activity of insulin-degrading enzyme (IDE), which normally breaks down Aβ, by generating excessive oxidative stress [297, 298]. Moreover, insulin resistance also lowers the expression of low-density lipoprotein receptor-related protein 1, a transporter responsible for moving Aβ across the BBB, leading to reduced Aβ clearance [299–301]. Furthermore, insulin resistance impairs the Aβ uptake and clearance functions of microglia and astrocytes [302, 303].

In addition to impairing Aβ clearance, insulin resistance also promotes Aβ production. It increases the expression of β-secretase 1 and activates GSK-3β, both of which are involved in the processing of APP into Aβ [304, 305]. In addition, the activation of GSK-3β also enhances tau phosphorylation, which is critical for the formation of tau tangles [306]. Insulin resistance further disrupts glucose metabolism and exacerbates mitochondrial dysfunction, leading to increased production of ROS and heightened oxidative stress [307–310]. This oxidative environment promotes the formation of toxic Aβ aggregates [311]. These findings highlight a strong mechanistic link between NDDs and diabetes [312]. Taken together, the underlying processes responsible for the characteristic pathologies of AD likely contribute to cognitive dysfunction in individuals with diabetes, regardless of any acceleration caused by diabetes itself. Therefore, etiological treatment of AD and other forms of dementia may be highly relevant to diabetes. Additionally, from the prevention perspective, individuals with T2D who are at an increased risk of developing dementia can be identified at an early stage using established risk scores.

### Cross-seeding among NDDs

**Fig. 6:**
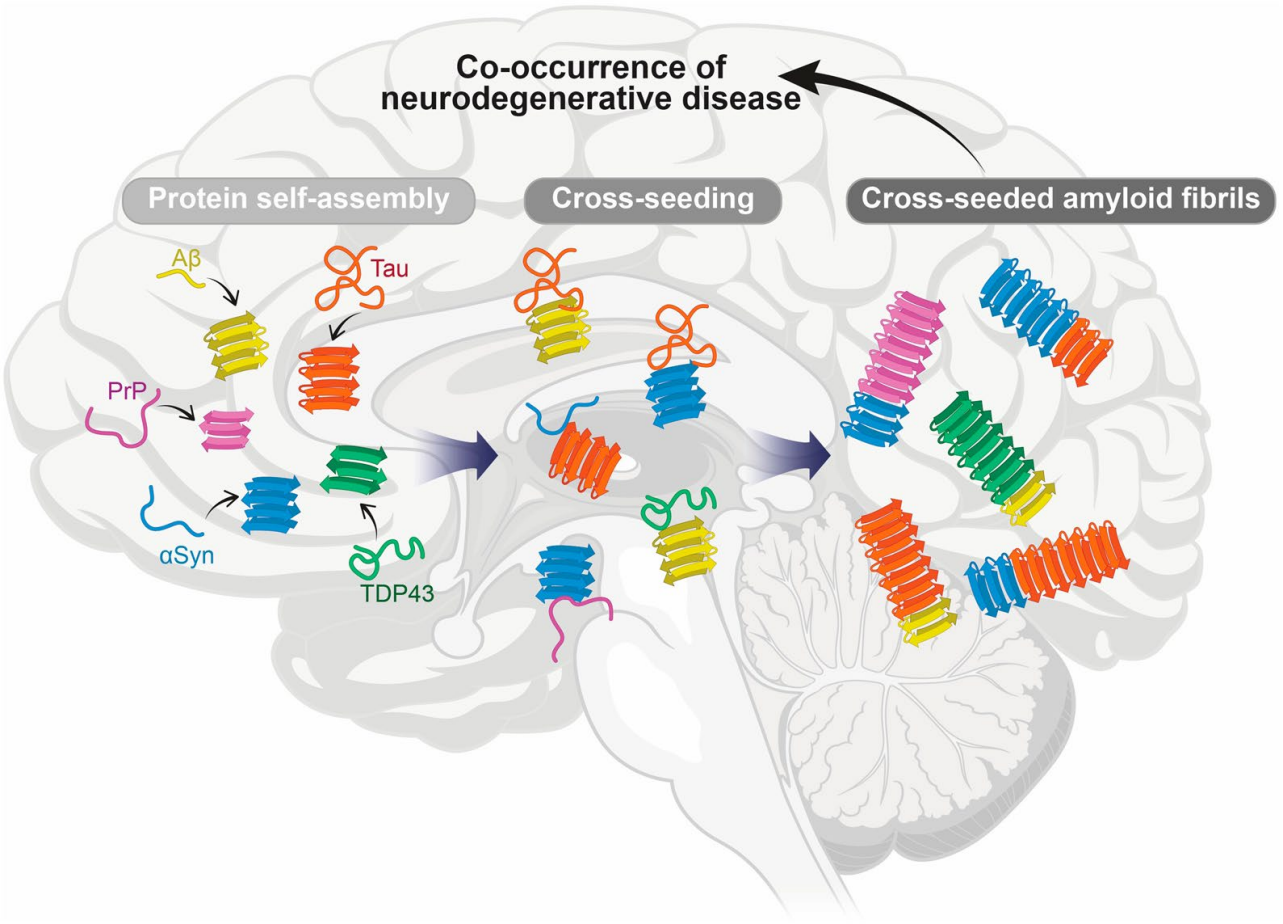
Schematic representation of cross-seeding events among amyloidogenic proteins in neurodegenerative diseases. Cross-seeding occurs when amyloid fibrils of one type induce the formation of amyloid fibrils of another type, playing a crucial role in neurodegenerative diseases. Observed cross-seeding events include: tau amyloid formation induced by Aβ amyloid fibrils [329, 330], tau amyloid formation induced by αSyn amyloid fibrils [345, 346], αSyn amyloid formation induced by tau amyloid fibrils [346, 347], TDP-43 amyloid formation induced by Aβ amyloid fibrils [11], and prion protein (PrP) amyloid formation induced by αSyn amyloid fibrils [11]. These cross-seeding interactions may contribute to the co-occurrence of multiple neurodegenerative diseases within the same patient

Most NDDs, including AD and PD, share common pathological characteristics and underlying mechanisms. One prominent molecular hallmark in many NDDs is the formation of amyloid aggregates composed of misfolded proteins, such as Aβ and tau in AD, αSyn in PD, TDP-43 in ALS, and prion protein in prion diseases. Recent investigations have revealed the occurrence of amyloid cross-seeding between different proteins (Fig. 6). This challenges the traditional view that NDDs are solely caused by the misfolding and aggregation of a single protein, suggesting significant overlaps among multiple disorders [11, 313, 314]. Post-mortem neuropathological analyses have further reinforced this perspective, consistently revealing the co-existence of multiple proteinopathies in the brains of individuals with NDDs. For instance, among 201 autopsied individuals with high levels of AD neuropathologic change, synucleinopathies were present in 55% of the cases, and TDP-43 proteinopathies were identified in 40% of the cases [315]. In another study involving 160 autopsy-confirmed cases of multiple system atrophy (MSA), 38% of individuals exhibited comorbid pathologies [316]. The most frequent was cerebral amyloid angiopathy (18%), followed by age-related tau astrogliopathy (9%), AD neuropathologic change (8%), and TDP-43 pathology (7%). These findings support the hypothesis that cross-seeding between distinct amyloidogenic proteins contributes to the heterogeneity and complexity of NDD progression.

Cryo-electron microscopy (cryo-EM) techniques have facilitated the determination of high-resolution structures of filamentous aggregates, improving our understanding of the cross-seeding activities [33, 317–320]. Despite variations in the amino acid sequences of amyloidogenic proteins, their fibrillar states share a common framework of cross-β sheet structure, which is considered crucial for amyloid cross-seeding [320]. Furthermore, the disordered regions of amyloid fibrils have been proposed to accelerate aggregation by recruiting monomeric proteins into the amyloid aggregates [321]. Additionally, misfolded protein aggregates may indirectly induce the aggregation of other pathological proteins by interfering with protein quality control systems, rendering cells more susceptible to misfolding and aggregation [322–324]. Despite significant progress, the molecular mechanisms underlying the cross-seeding of different amyloid proteins remain elusive. In this section, we illustrate key findings related to the phenomenon of cross-seeding among Aβ, tau, and αSyn.

#### Aβ and Tau

In AD, Aβ amyloid plaques typically accumulate outside cells, while tau forms NFTs within neurons, ultimately leading to widespread neuronal cell death [325]. The simultaneous presence of Aβ plaques and NFTs suggests a potential interconnection in the aggregation process of these two proteins [326]. Additionally, Aβ and tau have been found to co-localize intraneuronally and at synaptic terminals in the brains of individuals with AD, prompting extensive research into the phenomenon of cross-seeding between Aβ and tau [327–332].

Recent in vitro studies provide evidence supporting the accelerating effect of preformed Aβ aggregates on the fibrillar aggregation of tau [329, 330]. The presence of pre-aggregated Aβ significantly enhances the fibrillar aggregation of the microtubule-binding domain of tau (K18), compared to the control group [329]. Another study observed that Aβ amyloids can cross-seed the aggregation of full-length tau [330]. Computational studies have shown that Aβ amyloid fibrils can act as a sticky surface onto which tau fragments can bind, facilitating the formation of intermolecular β-sheets [333]. Consistently, direct interactions between K18 and both the self-recognition and C-terminal sites of Aβ have been identified [334]. This cross-seeding effect of Aβ amyloids on tau aggregation is believed to result from various interactions between Aβ and tau [335, 336]. Moreover, a peptide-based inhibitor designed to target Aβ aggregation effectively inhibited the aggregation and self-seeding of tau [330]. These findings suggest the existence of shared structural features between the core regions of Aβ and tau amyloids, which play a critical role in cross-seeding behaviour.

In vivo studies further enhanced our understanding of Aβ and tau cross-seeding. When preformed Aβ amyloids were injected into TauP301S transgenic mice, they efficiently induced tau aggregation and facilitated the propagation of tau pathology [331]. Additional evidence suggests distinct effects of Aβ isoforms on tau [332]. Experiments with transgenic flies and mice indicate that tau pathogenesis is promoted by the more aggregation-prone Aβ42 but not Aβ40 [332]. Furthermore, the introduction of tau in Tg2576 transgenic mice increases the expression of mutant APP and subsequent Aβ amyloid fibrillation [337]. These in vivo findings support the existence of cross-seeding interactions between Aβ and tau. Further studies on the high-resolution structures of the Aβ–tau complex could provide crucial information on key binding residues involved in the cross-seeding process, aiding in the development of effective therapeutic approaches for AD.

#### Tau and αSyn

αSyn is a presynaptic neuronal protein strongly associated with synucleinopathies, such as PD, dementia with Lewy bodies, and MSA. It undergoes misfolding and aggregation into oligomers and amyloid fibrils [338–341]. These aggregated forms contribute to progressive neuronal cell death by disrupting lipid membranes, impairing mitochondria, and deregulating calcium homeostasis [35, 340, 342, 343]. Notably, synucleinopathies exhibit clinical and pathological features similar to tauopathies, suggesting an interplay between tau and αSyn in their pathogenesis [344]. Recent studies have elucidated the pathological links between tau and αSyn, including potential cross-seeding events [345–347].

It has been reported that αSyn exhibits strong binding to the K18 region in tau, leading to the formation of hetero-oligomers [348, 349]. These oligomers possess high aggregation competence, accelerating the aggregation of both proteins and resulting in intraneuronal filaments [344]. Further research has demonstrated that preformed αSyn amyloid fibrils cross-seed the aggregation of tau, enhancing the formation of NFTs [350]. Preformed αSyn assemblies were reported to cross-seed tau fibrillar aggregation in a strain-dependent manner, suggesting that the structure of αSyn amyloids dictates the cross-seeding efficiencies on tau aggregation, subsequently leading to neurodegeneration [345, 346]. Cross-seeding events between αSyn and tau have also been observed in vivo. Utilizing mouse models, recent results have shown that tau and αSyn cross-seed each other [346]. Preformed αSyn amyloid fibrils accelerated tau aggregation, inducing robust tauopathy in PS19 mice [346]. Tau amyloids promoted αSyn pathology in M20 mice. Furthermore, cross-seeding between tau and αSyn has been shown to impair eyes and dopaminergic neurons in fruit fly models, highlighting its broader impact on their pathologies [347].

In addition to the direct cross-seeding effect, αSyn has been reported to indirectly promote tau aggregation by stimulating tau phosphorylation through PKA and glycogen synthase kinase-3β (GSK-3β) [351]. Despite recent advancements, the complex mechanism of cross-seeding and the pathogenic relationship between tau and αSyn remain poorly understood. Information regarding how post-translational modifications of proteins influence cross-seeding could be useful in identifying specific sites that modulate cross-seeding processes. Furthermore, the identification of high-resolution structures of αSyn–tau hetero-oligomers holds great promise for enhancing our understanding of the onset of NDDs and advancing the formulation of effective therapeutic strategies.

## Conclusion

Studies of disease-disease crosstalk have revealed shared pathological mechanisms and advanced our understanding of the ways in which distinct conditions influence each other’s progression. We explored the links between NDDs and seemingly unrelated conditions, such as infectious diseases, cancer, and T2D, emphasizing the molecular processes that underlie these interactions.

A key focus is the phenomenon of cross-seeding, where misfolded and aggregated proteins, including Aβ in AD, tau in tauopathies, and αSyn in PD, interact to promote further aggregation across diseases. For example, αSyn and tau can cross-seed each other’s aggregation, supporting a mechanistic basis for the frequent synuclein-tau comorbidity and compounded pathology in NDDs [352, 353]. Similarly, amyloidogenic proteins from pathogens, such as the spike protein of SARS-CoV-2 or SEVI in HIV, may influence amyloidogenesis in NDDs, although further investigations are required to establish this link [41, 92]. These insights underscore the broad relevance of cross-seeding in understanding the molecular basis of disease-disease interactions.

We also highlighted the inverse epidemiological associations between NDDs and cancer, alongside shared molecular mechanisms like p53 aggregation, which may offer dual-purpose therapeutic targets. The interplay between T2D and NDDs, exemplified by cross-seeding between amylin and Aβ aggregation, further illustrates how metabolic dysregulation can influence amyloid pathology and vice versa.

Drug repurposing has emerged as a promising strategy for developing new treatments by redirecting existing, clinically approved drugs to target different diseases and conditions [22]. There has been growing interest in applying this approach to NDDs [354, 355], but further progress requires a clearer understanding of the molecular mechanisms involved in disease-disease interactions. This knowledge can provide a foundation for identifying effective repurposing candidates for NDDs. To advance these efforts, future research should prioritize detailed investigations into the molecular mechanisms of cross-seeding, particularly through cryo-EM and other advanced structural techniques. Additionally, integrated systems biology approaches and large-scale clinical studies are needed to translate these findings into actionable therapies.

The recognition that distinct pathological conditions may intersect through shared molecular mechanisms offers an opportunity to reframe our understanding of chronic disease. By examining the common pathways that underlie neurodegeneration, malignancy, metabolic dysfunction, and infection, we are beginning to discern a network of biological interactions shaped not by chance but by the fundamental physicochemical constraints of cellular systems. These connections highlight the need for integrative approaches that move beyond traditional disease classifications. As our mechanistic knowledge deepens, therapeutic strategies that target core processes at the intersection of multiple diseases can be potentially developed. Such approaches, especially when rooted in molecular precision, may ultimately allow us to modulate the progression of complex disorders not in isolation, but within the broader context of the physiological landscape in which they arise.

## Data Availability

Not applicable.

## Abbreviations

Aβ: Amyloid-β
ACE: Angiotensin-converting enzyme
AD: Alzheimer’s disease
AIDS: Acquired immunodeficiency syndrome
ALS: Amyotrophic lateral sclerosis
APOE: Apolipoprotein E
APP: β-Amyloid precursor protein
αSyn: α-Synuclein
BBB: Blood–brain barrier
BZLF1: BamHI Z fragment leftward open reading frame 1
CMA: Chaperone-mediated autophagy
CNS: Central nervous system
cryo-EM: Cryo-electron microscopy
EBV: Epstein–Barr virus
EBNA1: Epstein–Barr nuclear antigen 1
GLUT: Glucose transport
GSK-3β: Glycogen synthase kinase-3β
HD: Huntington's disease
HIF-1α: Hypoxia-inducible factor-1α
HIV: Human immunodeficiency virus
HSV-1: Herpes simplex virus type-1
mHTT: Mutant huntingtin
MS: Multiple sclerosis
MSA: Multiple system atrophy
mTOR: Mammalian target of rapamycin
NDD: Neurodegenerative disease
NLRP3: Nucleotide-binding domain leucine-rich repeat, and pyrin domain containing receptor protein 3
Nrf2: Nuclear factor erythroid 2-related factor
ORF: Open reading frame
PAP: Prostatic acid phosphatase
PD: Parkinson's disease
PI3K: Phosphoinositide 3-kinases
PKA: Protein kinase A
PKC: Protein kinase C
RBPs: RNA-binding proteins
ROS: Reactive oxygen species
SEVI: Semen-derived enhanced of viral infection
siRNA: Small interfering RNA
T1D: Type 1 diabetes
T2D: Type 2 diabetes
TDP-43: TAR DNA-binding protein 43
